# Test of *Arabidopsis* Space Transcriptome: A Discovery Environment to Explore Multiple Plant Biology Spaceflight Experiments

**DOI:** 10.3389/fpls.2020.00147

**Published:** 2020-03-04

**Authors:** Richard Barker, Jonathan Lombardino, Kai Rasmussen, Simon Gilroy

**Affiliations:** ^1^ Department of Botany, University of Wisconsin, Madison, WI, United States; ^2^ Microbiology Doctoral Training Program, University of Wisconsin, Madison, WI, United States

**Keywords:** *Arabidopsis thaliana*, spaceflight, transcriptomics, RNAseq, microarray, proteomics, bioinformatics, Qlik

## Abstract

Recent advances in the routine access to space along with increasing opportunities to perform plant growth experiments on board the International Space Station have led to an ever-increasing body of transcriptomic, proteomic, and epigenomic data from plants experiencing spaceflight. These datasets hold great promise to help understand how plant biology reacts to this unique environment. However, analyses that mine across such expanses of data are often complex to implement, being impeded by the sheer number of potential comparisons that are possible. Complexities in how the output of these multiple parallel analyses can be presented to the researcher in an accessible and intuitive form provides further barriers to such research. Recent developments in computational systems biology have led to rapid advances in interactive data visualization environments designed to perform just such tasks. However, to date none of these tools have been tailored to the analysis of the broad-ranging plant biology spaceflight data. We have therefore developed the Test Of Arabidopsis Space Transcriptome (TOAST) database (https://astrobiology.botany.wisc.edu/astrobotany-toast) to address this gap in our capabilities. TOAST is a relational database that uses the Qlik database management software to link plant biology, spaceflight-related omics datasets, and their associated metadata. This environment helps visualize relationships across multiple levels of experiments in an easy to use gene-centric platform. TOAST draws on data from The US National Aeronautics and Space Administration’s (NASA’s) GeneLab and other data repositories and also connects results to a suite of web-based analytical tools to facilitate further investigation of responses to spaceflight and related stresses. The TOAST graphical user interface allows for quick comparisons between plant spaceflight experiments using real-time, gene-specific queries, or by using functional gene ontology, Kyoto Encyclopedia of Genes and Genomes pathway, or other filtering systems to explore genetic networks of interest. Testing of the database shows that TOAST confirms patterns of gene expression already highlighted in the literature, such as revealing the modulation of oxidative stress-related responses across multiple plant spaceflight experiments. However, this data exploration environment can also drive new insights into patterns of spaceflight responsive gene expression. For example, TOAST analyses highlight changes to mitochondrial function as likely shared responses in many plant spaceflight experiments.

## Introduction

As a possible integral feature of life support systems, plants offer the potential to provide food, replenish the air, filter water, and improve the mental health of the crew during long-duration missions in space. Therefore, at a practical level, plants are being intensively studied to assess their ability to adequately fulfill these roles in the spaceflight environment [reviewed in (Wheeler, 2017)]. In addition, a growing number of plant spaceflight studies are addressing the quest for fundamental knowledge about how plant biology operates. Thus, the spaceflight environment provides conditions that are inaccessible on Earth, such as growth in microgravity and exposure to cosmic radiation, providing a unique opportunity to dissect responses under conditions that plant biology has not encountered during its evolutionary history [reviewed in (Vandenbrink and Kiss, 2016; Paul et al., 2013a)].

These studies are now generating extensive characterizations of the responses of diverse plant species to spaceflight. As part of the output from this research, there is an ever-increasing set of genome-scale analyses that range from transcriptomics [e.g., (Kwon et al., 2015; Johnson et al., 2017; Paul et al., 2017; Choi et al., 2019; Herranz et al., 2019; Vandenbrink et al., 2019)] and proteomics [e.g., (Mazars et al., 2014; Ferl et al., 2015; Basu et al., 2017)] to epigenomics [e.g., (Zhou et al., 2019)]. These datasets help catalog the plant response to growing in space. For example, the omics database maintained by The National Aeronautics and Space Administration’s (NASA’s) GeneLab program (GeneLab, 2019) contains, at the time of writing, data from over 200 spaceflight-related experiments, with about 20 plant-focused studies, mainly from research conducted using the Space Shuttle and International Space Station. In addition, similar spaceflight and related data from e.g., the Japanese, Chinese, and European space agencies have been deposited in a range of other publicly accessible data repositories such as NCBI GEO (Barrett et al., 2013) and the European CATdb (Gagnot et al., 2008). Each experiment has multiple spaceflight samples and often compares the responses of wild-type and mutants in spaceflight to parallel ground-based controls performed on the Earth. Research groups have then mined, e.g., the patterns of transcriptional change seen in individual experiments to reveal potential underlying plant responses to spaceflight. Thus, changes in the expression of heat shock proteins [e.g., (Zupanska et al., 2013; Johnson et al., 2017; Li et al., 2017; Choi et al., 2019)], cell wall peroxidases [e.g., (Correll et al., 2013; Kwon et al., 2015; Zhang et al., 2015; Johnson et al., 2017; Choi et al., 2019)], and a general response to oxidative stress [e.g., (Sugimoto et al., 2014; Choi et al., 2019)] have all emerged as response signatures identified in some, but not all, plant spaceflight transcriptomes. However, the scale of the available data now poses challenges when making such comparisons between diverse experiments. Thus, (1) the datasets are distributed across multiple repositories, posing potential issues with accessibility and interoperability, (2) the bioinformatics-based analytical approaches used between published studies are often very different, making robust comparisons of differences drawn from the literature challenging, (3) the sheer scale of the data makes it hard to perform more than a few comparisons between experiments before its volume becomes limiting, and (4) it is often difficult to present these kinds of broad-scale comparative analyses in a visually accessible, intuitive manner for use by a broad scientific audience.

To address these challenges, we have developed the Test Of Arabidopsis Space Transcriptome (TOAST; a compilation of the abbreviations and terms used throughout, along with a brief definition of each is presented as a glossary in the **Appendix**) database. TOAST uses a database management software called Qlik (Qlik Technologies Inc., King of Prussia, PA, USA) to aggregate and visualize plant spaceflight omics-level data from multiple repositories. It applies a uniform set of analytical steps to the data and makes visualization of massive datasets accessible, allowing for interactive comparisons between experiments. The database also provides links to experiment metadata and a suite of online tools to enhance the scope of potential further analysis. In this publication we present an overview of the TOAST database and provide examples of how it can both validate previously published inferences as to likely spaceflight-imposed stress responses and mine across the plant spaceflight transcriptomics data to facilitate the generation of new hypotheses.

## A Broad Set of Available Data Underlies the TOAST Data Exploration Environment

**Figure 1:**
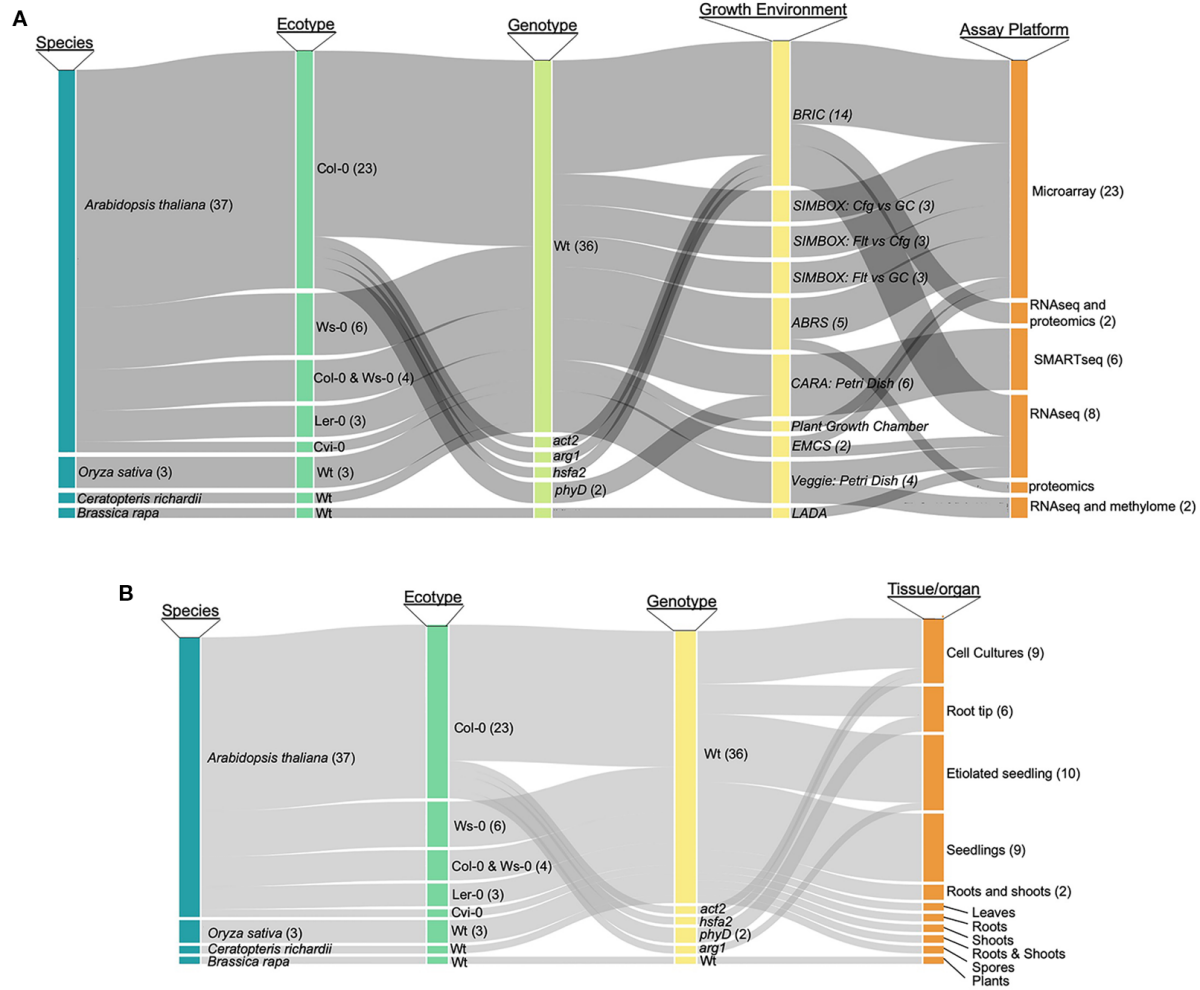
Publicly available spaceflight transcriptomics datasets. **(A)** Relationships between species, ecotype, genotype (i.e., mutant or wild type) growth environment and assay technique for datasets from plants experiencing spaceflight. **(B)** Relationships between species, ecotypeand genotype versus the tissue or organ type that was sampled to generate the tspaceflight-related transcriptomics dataset. Col-0, Columbia ecotype of *Arabidopsis thaliana*; Ws, Wassilewskija ecotype; Ler, Landsberg erecta ecotype; Cvi, Cape Verdi Islands ecotype; mutants of Arabidopsis: *phyD*, Phytochrome D; *arg*, Altered Response to Gravity; *act2*, Actin 2; *phyD*, phytochrome D; *hsfa2*, heat shock factor a2, Wt, wild type; BRIC, Biological Research in Canister; ABRS, Advanced Biological Research System; EMCS, European Modular Cultivation System.

As a first step toward designing a comprehensive tool for the analysis of plant spaceflight omics-level data, we categorized the breadth of data available to support such an exploration environment. As most studies have generated transcriptomics data, we have focused on these datasets, although the TOAST database also includes the currently few available proteomic (Mazars et al., 2014; Ferl et al., 2015; Basu et al., 2017) and epigenomic (Zhou et al., 2019) plant spaceflight datasets. NASA's GeneLab program maintains a publicly accessible data repository that brings together a large amount of such genome-scale spaceflight data (GeneLab, 2019). Although the GeneLab site has the highest density of these kinds of spaceflight-related datasets, the global spaceflight research community has deposited a large amount of data generated by similar genome-scale experiments in other data repositories such as NCBI-GEO (Barrett et al., 2013) and CATdb (Gagnot et al., 2008). **Figure 1** presents an analysis of the spectrum of plant species and experimentation available for incorporation into a plant-focused data exploration environment (see **Supplementary Table 1** for the source list of the plant biology data repositories). The most highly researched plant is *Arabidopsis thaliana*, being the predominant plant model for molecular analysis, with the Col-0 ecotype most frequently chosen for spaceflight experimentation. Rice [*Oriza sativa*; (Jin et al., 2015)], mizuna [*Brassica rapa*; (Sugimoto et al., 2014)], and the fern *Ceratopteris richardii* (Salmi and Roux, 2008) have also been the focus of similar molecular analysis. **Figure 1A** breaks down the available data into species and genotype versus analytical approach (e.g., microarray or RNAseq technologies), showing that the majority of the available data has been generated using Affymetrix microarrays or Illumina-based RNAseq to monitor patterns of gene expression. **Figure 1B** further shows that although the predominant sample analyzed in multiple plant spaceflight experiments is the whole seedling, data is available from several experiments using cell cultures and from individual organs dissected after the plants had been grown in space.

This survey of the available plant biology spaceflight-related data suggested to us that there is a strong base of publicly accessible, genome-level datasets with which to populate a database designed to help visualize and compare between plant spaceflight experiments. For example, there are multiple experiments using similar species and analyzing similar tissues; transcriptomics data for *Arabidopsis* is particularly extensive. We set a minimum criterion for inclusion in the initial iteration of TOAST to be studies where statistically rigorous analyses can be applied. This approach means datasets are required to contain three or more biological replicates and at present only spaceflight experiments on *Arabidopsis* and rice fulfill this requirement. We have therefore imported all of the available, replicated *Arabidopsis* and rice plant spaceflight datasets into the TOAST database. In addition, we have added a series of ground-based datasets addressing spaceflight-related factors, such as effects of increased radiation or exposure to oxidative stress on Earth as the foundation with which to build the TOAST exploration environment (these datasets are summarized in **Supplementary Table 1**).

## TOAST Design Philosophy and Data Structure

As noted above, the underlying software engine behind TOAST is the Qlik Associative Engine (Qlik Technologies Inc., King of Prussia, PA, USA). We chose to use Qlik as it not only provides the tools to develop and administer the underlying relational database but also allows the user to readily see what other information in the database is associated with their current query *via* a software feature built into QLIK named the Qlik Associative Data Engine. In addition, Qlik integrates graphic visualization packages that allow intuitive, interactive exploration and analysis of the data. Such tools help ensure the data will be more readily accessible not only to plant space biology researchers and bioinformaticians but also to a much broader community, including non-specialists and students.

**Figure 2:**
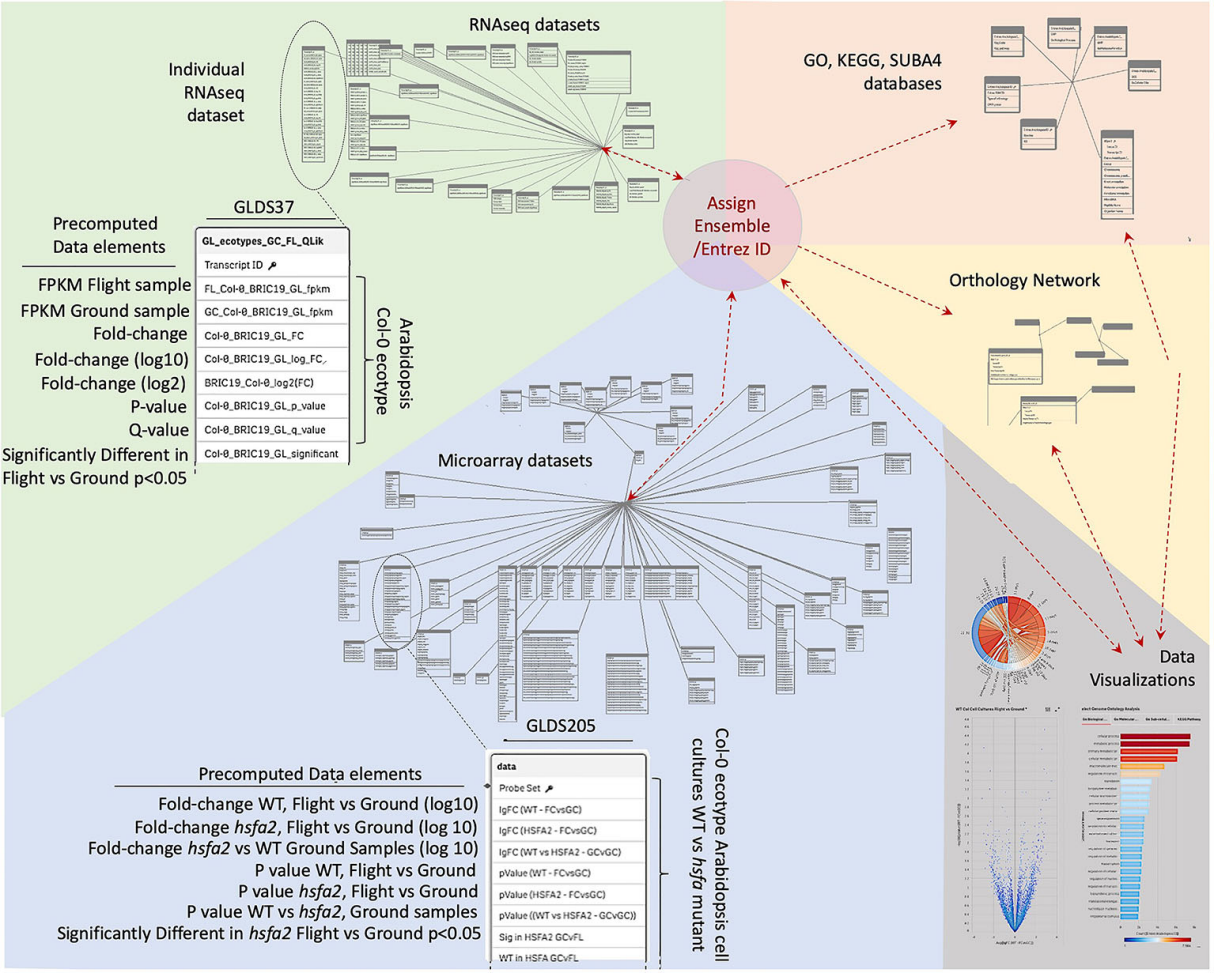
Database structure underlying TOAST 4.5. Each dataset within TOAST includes a series of pre-computed factors for each gene: minimally including fold-change, P-value, Q-value, and a yes/no value for whether the fold-change for each gene is significant at P < 0.05. These pre-computed values greatly speed the real-time processing of interactive visualizations within the TOAST user interface. The identifiers in the raw data, such as Transcript ID from RNAseq, Probe ID for Microarray, or TAIR ID are translated to their unique Entrez and Ensembl IDs to allow for uniform indexing within TOAST itself and to facilitate passing of analyzed data produced by TOAST analyses to exterior sites and tools. Within TOAST, the strings of molecular ID's from a dataset are both directly transferred to a series of data visualization and exploration tools and are imported into a series of analytical packages accessing a range of databases that have been imported into the TOAST environment. These databases include: the Genome Ontology (GO) consortium databases that allow analysis of the relationships between gene lists of interest and known biological processes, the SUBA4 database which catalogs predicted subcellular locales for each gene, the Kyoto Encyclopedia of Genes and Genomes (KEGG) database that analyzes relationships to known cellular pathways, and Ensembl's Orthologous Matrix database, allowing TOAST to make comparisons between species. The outputs of these analytical modules are then passed to TOAST's interactive data visualization tools to help explore each dataset. Results from the visualizations are in turn returned as lists of Gene IDs to allow for reiterative analyses.

Data was therefore imported into a Qlik-managed database (**Supplementary Table 1**) to generate the associative database outlined in **Figure 2** that forms the foundation of TOAST functionality. However, the various data sources use a variety of indices for gene identification that range from Affymetrix microarray probe name (i.e., the Affymetrix microarray technology's specific technical name for the DNA probe used to identify a particular gene) to Arabidopsis Genome Initiative (AGI) locus codes [i.e., unique gene identifiers assigned by the consortium of researchers forming the Arabidopsis Genome Initiative; (Kaul et al., 2000)]. We therefore first re-indexed all the datasets to use Entrez gene identifiers (Maglott et al., 2011). Entrez is the National Center for Biotechnology Information (NCBI)'s database for gene-specific information and it assigns gene identifiers, or codes, that uniquely identify a particular gene. The advantage of these identifiers in tracing a gene from one dataset to another is that they form a uniform, well-curated indexing system specifically developed to be applied across all organisms. Entrez gene names uniquely identify individual genes and importantly, the system has been developed to expand as new genes are identified. Thus, re-indexing the gene identifiers in TOAST from the varied standards used in the imported datasets to their Entrez identifiers served several purposes: (1) it allows for comparisons within the TOAST database *via* a uniform labeling system, (2) it facilitates data exchange with other databases and analytical tools, anchoring the data to the global Entrez standard, and (3) it builds scalability into the database architecture as Entrez identifiers are designed to provide a standard for indexing all current and future documented genes.

If the original authors' data structure matched that of our database model (minimally, fold-change, P-value, Bonferroni corrected Q-value), we imported their analysis that incorporated their statistical models for calculating P- and Q-values. However, some of the publicly available microarray dataset analyses lacked some of these minimum requirements (most typically missing a statistical analysis of significance of reported changes) and so had to be reprocessed for incorporation into TOAST. For this reanalysis (exclusively Affymetrix ATH1 microarray data), we used R-studio codes provided courtesy of NASA's GeneLab. R is a programming language widely used in the statistical analysis of scientific data (https://www.r-project.org/about.html) and R-studio is commercially produced software that aids with the development of programs using R (R-Studio Inc. Boston, MA, USA). These R-studio codes were customized for each experimental design to provide the required fields of fold-change, P-, and Bonferroni corrected Q-values using Robust Multichip Average (RMA) quantile normalization (Irizarry et al., 2003), a technique that accounts for variation across multiple microarray chips used in these analyses. These codes can be found at https://github.com/dr-richard-barker/NASA-GeneLab-MicroArray-Codes. Further analysis was then performed on the imported data from Arabidopsis Affymetrix and CATMA microarrays and rice Affymetrix microarrays, converting probeIDs to Entrez gene identifiers. RNAseq data was reprocessed by importing the raw FASTQ files (i.e., the files containing the nucleotide sequences identified by the sequencing machine) into CyVerse [the cloud computing infrastructure supported through the National Science Foundation's Directorate of Biological Sciences; (Merchant et al., 2016)] and then analyzed using a series of software steps (analysis pipeline) of: HiSAT to first generate BAM files from the FASTQ files. BAM, or Binary compressed sequence Alignment Map files contain information on the alignment of each read from the sequencing machine to the genome. BAM files were then processed by the BAMtoCounts software package to create a counts matrix that holds the number of reads that have mapped to a particular transcript. Finally differential expression analysis for each transcript was calculated using the DESeq/EdgeR approach (Love et al., 2014) as part of the iDEP R-Shiny application. iDEP is a software package for the R programming language designed to process genetic data. iDEP uses R-Shiny, a further R software package that allows for easy development of interactive web-based applications (Ge et al., 2018). Fragments per kilobase of transcript per million mapped reads (FPKM) and counts per million reads mapped (CPM) were calculated as described in Choi et al. (2019).

We used the TAIR10 annotation that describes genetic loci within the *Arabidopsis thaliana* genome sequence (Lamesch et al., 2012) and the associated genes were linked to Gene Ontology (GO) molecular function and biological processes databases that catalog the annotated functions and processes linked with each genetic locus. These GO descriptions allow testing of whether genes annotated as being associated with specific molecular functions or biological processes are over represented in a particular dataset (Ashburner et al., 2000; Carbon et al., 2019). Consensus sub-cellular location predictions were imported from the SUBA4 subcellular locale database (Hooper et al., 2017). SUBA4 uses multiple weighted lines of empirical evidence for protein localization in addition to aggregating subcellular targeting predictions from >20 programs, providing a broad-scale survey of likely subcellular association for the protein product of each transcript. As these databases use a variety of gene identifiers for their indexing, a table was developed within the Qlik database to translate between these various identifiers and the Entrez indexing within TOAST. This matrix linked the TAIR AGI with the associated Affymetrix microarray Probe IDs, RNAseq transcript IDs, along with associated Ensembl ID [imported from the Ensemble BioMart plant database; (Zerbino et al., 2018)], Entrez ID and if available, the Gene Symbols (i.e., the commonly used gene name). Rice cell culture microarray results from the Shenzhou 8 mission (Kindly provided by Dr. Peipei Xu, Shanghai Institute of Plant Physiology and Ecology and Dr. Weiming Cai, Chinese Academy of Sciences) were also integrated into TOAST. To allow comparison to the Arabidopsis data, we adopted an orthologous matrix (OM) database-driven approach. Thus, the Ensembl genome database project (Kersey et al., 2018) has developed software to analyze the structure of the genomes of different organisms to identify genes between species that originated from a common ancestral gene prior to speciation [i.e., orthologous genes; (Altenhoff et al., 2018)], allowing researchers to ask if, e.g., transcriptome responses between species reflect similar patterns of classes of gene expression. To allow such comparisons within the TOAST database, we needed to be able to translate rice microarray probe IDs to orthologous Arabidopsis gene identifiers. We therefore imported the OM table from Ensembl, i.e., the table that links the rice and Arabidopsis orthologs through their Ensembl gene IDs. We then linked the rice microarray probe IDs provided in the imported rice datasets within this table to their corresponding Ensemble IDs, allowing mapping between the *Arabidopsis* and rice orthologs.

## The TOAST User Interface

**Figure 3:**
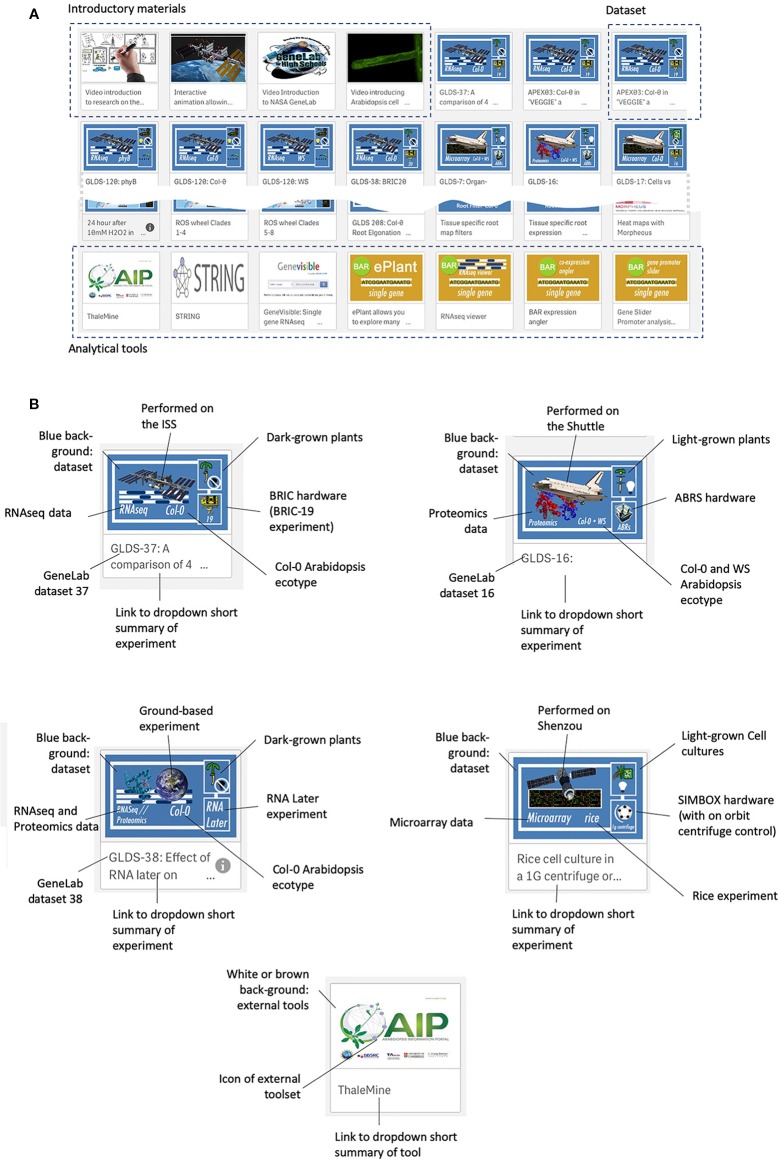
The TOAST 4.5 user interface. **(A)** The web interface for TOAST launches an overview menu of dashboard icons allowing the user to directly access the introductory materials, omics data, and related analysis tools. **(B)** Each icon provides a visual summary of the data or tools that it links to including elements such as spaceflight vehicle (e.g., Shuttle, ISS, Shenzou vs ground-based experimentation), the growth hardware used, plant/seedling vs cell culture experiment, RNAseq vs microarray vs proteomics, species and ecotype and dataset identifier (e.g., GLDS number).

**Figure 3** shows the web interface for TOAST, which launches as an overview menu of dashboard icons. Clicking on the first few dashboards links to introductory materials about space or Arabidopsis research providing an entry point for the non-specialist. Most of the remaining icons are links to datasets from individual experiments. The design of each icon gives quick visual information on the nature of the experiment (spacecraft, plant type, hardware, assay type) and clicking on the icon opens the particular dataset. The final set of icons represent links to online tools that can be used to further analyze the results emerging from using the TOAST database. The linked tools are summarized in **Supplementary Table 2**.

**Figure 4:**
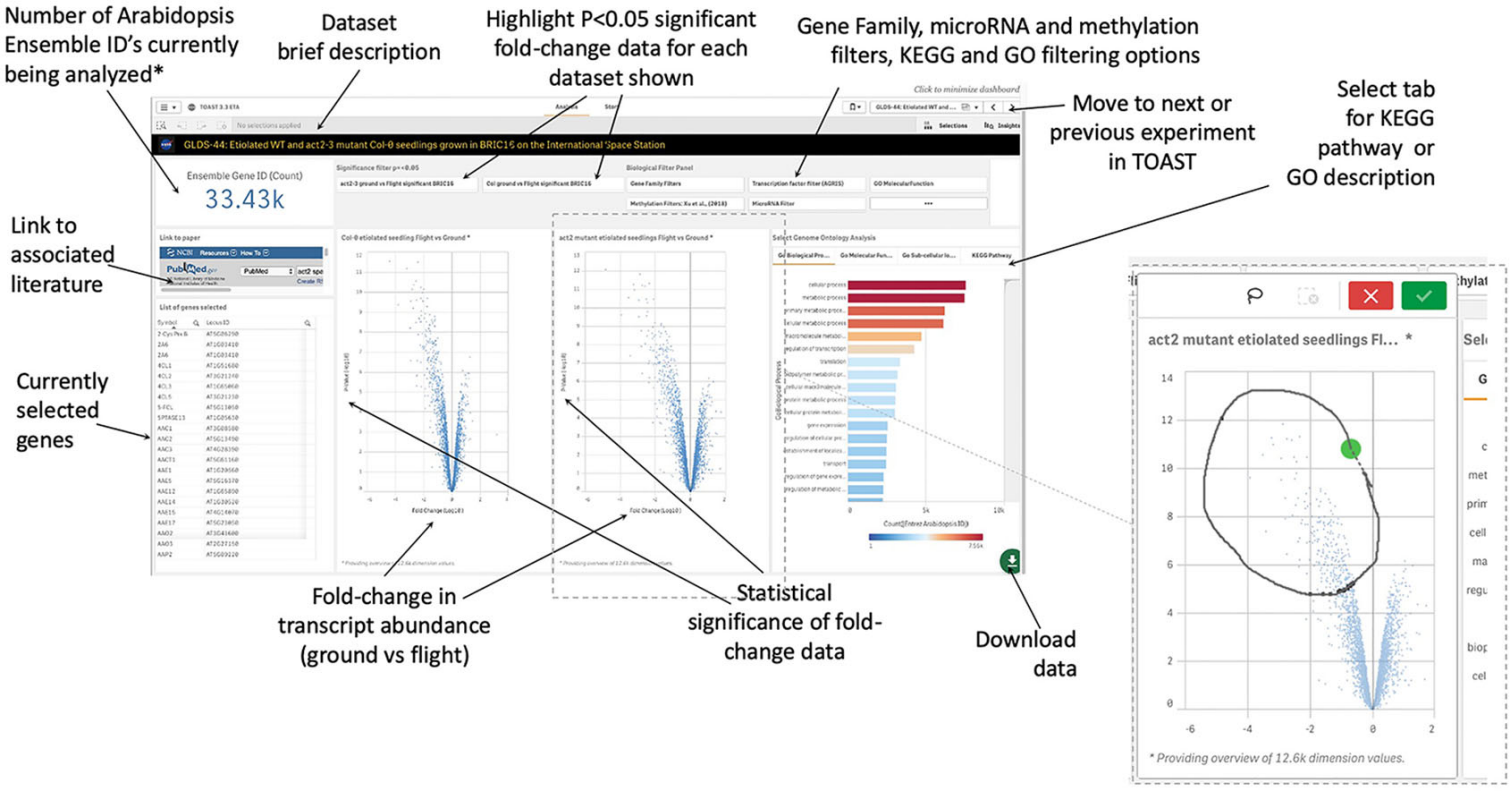
Graphical user interface for typical dataset. Clicking on a volcano plot also activates an interactive graphical tool for manual selection of groups of genes of interest. *Defaults to showing 33.43K, i.e., all Entrez identifiers, until a filter or gene selection is applied. Inset, a lasso tool allows user selection of data points from volcano plot in addition to activation of filters such as on significance of change, KEGG Pathway, or GO annotation.

Within TOAST, each dataset is presented to the user as an interactive dashboard with Log10-fold change and measure of statistical significance (P-value) provided for each locus as shown in **Figure 4**. We have used P- rather than Q-value as our initial metric of significance to provide as broad an overview of significantly differentially expressed genes as possible. Q-values are corrected P-values that take into account the cumulative errors that occur when making multiple tests of significance within a large dataset. The Q-value is available in the downloadable data tables (see below), allowing users to apply this more stringent statistical metric as needed. Volcano plots (plots of fold-change versus statistical significance of that change for each gene ID) were chosen as the main way to visualize both the statistical analysis and the degree of gene induction or repression. A side table displays the gene identifier, the gene symbol, the fold-change and P-value and Q-value for each locus. The user can toggle on and off the P-value statistical significance filters on the volcano plot to rapidly assess the strength of the inferences to be drawn from the results that they are visualizing. All of these data can also be downloaded and used with a range of other databases that are linked in the TOAST overview menu (**Figure 3**). TOAST 4.5 includes a GO database (Ashburner et al., 2000; Carbon et al., 2019) that provides real time feedback on the ontology of the subsets of genes selected. Tabs above the interactive bar charts allow access to four main types of annotation: GO Molecular function (16,504 categories), GO Cellular component (15,383 categories), GO Biological process (15,644 categories), and Kyoto Encyclopedia of Genes and Genomes pathways [KEGG; (Kanehisa et al., 2017)]. KEGG is a widely used database that categorizes genes into the cellular pathways in which they are involved. In addition, gene selections can be interactively compared against the AGRIS transcription factor database [1,851 loci; (Palaniswamy et al., 2006)], the TAIR10 microRNA database (Lamesch et al., 2012), or be filtered using a selection of over 60 manually curated gene families. A further selection allows comparison to known sites of spaceflight-induced epigenetic modification (Zhou et al., 2019). These filters are applied using a drop-down menu. NCBI PubMed links to any associated publication are also embedded alongside these data analysis tools to provide the critical context of the original published experimental descriptions and analyses (a summary of literature linked within TOAST is shown in **Supplementary Table 1**).

**Figure 5:**
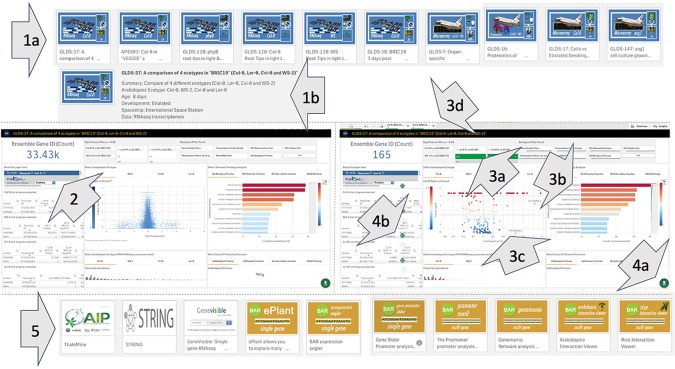
Overview of use of the TOAST 4.5 database. (1a) The user selects an initial study of interest and then can review the summary of its metadata to ensure it is the correct focus for study (1b). The dataset is then opened and (2) when the study is selected an interactive dashboard launches and the user has a direct link to any associated manuscript. Gene filtering: statistical (3a), gene ontology (3b), and other related functional filters can be applied to focus the number of loci being visualized in the volcano plot (3c) to genes of interest. In addition, the volcano plot itself can be interactively manually filtered using a graphical selection tool. All filters can be toggled on and off using selectable tabs at the top of the interface (3d). If an interesting subset of loci are selected the user can activate the download option (4a) and save the related data in word or xml format (4b). (5) The user can also perform further bioinformatic and statistical analysis with other online tools linked from the main user interface.

These data exploration features were implemented using D3 JavaScript software libraries executed within the Qlik environment, connecting the spaceflight data, its pre-computed statistics (**Figure 2**) and information on functional ontology. This system architecture facilitates user interaction with massive amounts of data in real time. Thus, as shown in **Figure 5**, the user selects a dashboard containing their initial dataset of interest. The software then allows them to interactively select genes or groups of genes either manually from the volcano plots, by filtering using gene ontology terms, or *via* a text-based interface as described above. As the user explores the data, they can apply further rounds of filtering and/or manual selection of groups of genes. These stacked filters spawn to all other datasets such that opening another dashboard of information on another experiment will show the equivalently filtered results. Further filtering of these newly opened data will, in turn, filter back on the original and all other datasets. This reiterative filtering approach allows the user to focus on an ever smaller number of genes selected by comparisons across multiple experiments. These results can be exported as a spreadsheet and/or passed to other web-based analytical sites linked within the TOAST interface.

## TOAST Metadata App

**Figure 6:**
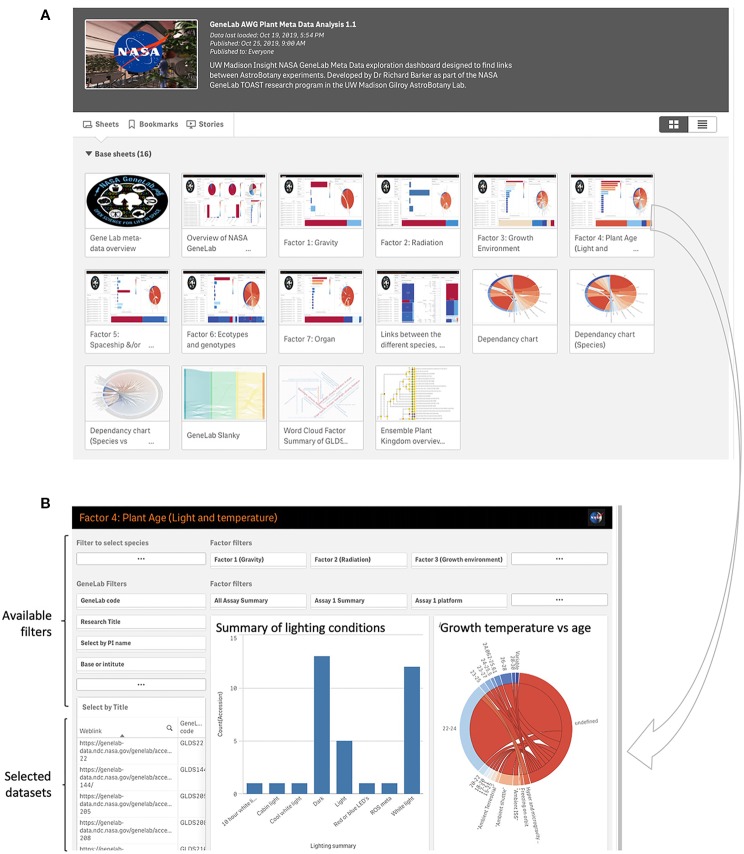
Analysis of metadata within the TOAST 4.5. **(A)** Initial dashboards allow access to comparisons between a range of experiment-related factors such as lighting conditions, growth environment, and plant genotypes. **(B)** A typical dashboard for metadata exploration, in this case for light conditions and age of seedling. Preset filters for e.g., lab group performing the research and growth and radiation environments are available to the user and the identity of the filtered datasets is shown in the bottom left window.

A custom metadata app is also incorporated as a tool for use with TOAST (https://astrobiology.botany.wisc.edu/astrobotany-toast/tutorial-metadata). This additional relational database provides data visualization tools that use the metadata associated with each dataset to find associations in factors such as experimental design parameters, hardware, and features of the spaceflight mission between different experiments in TOAST 4.5. GeneLab provides a rich array of metadata associated with its datasets. Most of the other non-GeneLab datasets incorporated into TOAST do not provide these kinds of metadata summaries and so we manually curated both the GeneLab and non-GeneLab datasets within TOAST 4.5 to provide equivalent metadata for all. These experiment-related factors are presented in **Supplementary Table 3** and drive the visualizations presented in the metadata app. **Figure 6A** shows the main dashboard for the metadata app. Clicking on an icon launches the associated dashboard where interactive visualizations can, in turn, filter on the range of factors that are presented (**Figure 6B**). This architecture allows the user to explore commonalities in the available plant biology data in TOAST 4.5 ranging from lighting conditions, hardware or plant age at time of assay to analytical approach and even PI of the group performing the experiment (See **Supplementary Table 3** for comprehensive list of factors). Within the app there are several places where “ROS meta-analysis variable” appears on the visualization. This description is used to denote that the data comes from a published meta-analysis of many publicly available microarray experiments related to responses to reactive oxygen species (ROS) called “The ROS-wheel” (Willems et al., 2016). Thus, for this particular comparative dataset there is not a single value of, e.g., for light level or plant age (as it is an aggregation of many individual experiments).

## Overview of the Plant RNAseq and Microarray Data Within TOAST 4.5

For the RNAseq data in TOAST, 42,220 transcript IDs are assigned to one of 37,019 distinct TAIR10 gene models. However, only 33,550 transcripts were detected within the data imported into TOAST 4.5 as being expressed either on Earth or during spaceflight. For microarrays, TOAST 4.5 links data gathered from 22,810 Arabidopsis Affymetrix probes IDs, 7,370 CATMA probe IDs, and 75,070 rice probe IDs. For Arabidopsis, the 42,220 Entrez loci ID's are associated with 13,750 detected proteins and, if it has been assigned, to one of the 25,270 Gene Symbols [drawn from the TAIR and ATTED II (Obayashi et al., 2018) gene databases combined]. For rice, 75,000 Affymetrix probe IDs are linked to the Arabidopsis Ensembl ID as described above. Note, in some microarrays a subset of the probes used have the potential for cross-hybridization and so to report on multiple gene responses. Similarly, many microarrays have redundant probes for each gene (e.g., in addition to gene unique probes, the ATH1 microarray also has 309 probes that redundantly monitor 148 genes). Where we have imported the original authors' analyses, we have used their approach to identifying and filtering these effects. When we had to reanalyze a dataset to conform to our requirements of presenting fold-change and P- and Q-values, then where a probe was identified as showing potential cross-hybridization effects, we have assigned a gene ID with both gene identifiers. Thus, e.g., a data point derived from a potentially cross-hybridizing probe represented on a volcano plot of fold-change versus significance would simultaneously show both gene IDs. For redundant probes, it is known that these often do not agree on expression levels (Cui and Loraine, 2009). This is likely due to the fact that each probe hybridizes to a different point on the gene and so effects such as differential splicing of that gene will cause probes to behave differently in the gene expression analysis. To be as inclusive as possible, the maximum value amongst each redundant probeset was therefore used.

Most of the microarray data within the TOAST database is associated with the Affymetrix ATH1 chip, with Illumina-based RNAseq being the second most regularly used approach. For these experiments, *Arabidopsis* seedlings were grown under a range of growth hardware and lighting conditions. Experiments in the Biological Research in Canisters (BRICs) produced dark-grown samples in cassettes (Petri dish fixation units, PDFUs) that are sealed prior to launch [e.g., (Kwon et al., 2015; Basu et al., 2017; Johnson et al., 2017; Zupanska et al., 2017; Choi et al., 2019)]. Light-grown material was produced in the European Modular Cultivation System [EMCS, with variable RGB lighting and atmospheric and temperature control, e.g., (Correll et al., 2013; Herranz et al., 2019; Vandenbrink et al., 2019)], in SIMBOX [Science in Microgravity Box, LED lighting, e.g., (Fengler et al., 2015)], and in Petri dishes under 24 h LED light in the Veggie hardware [e.g., (Beisel et al., 2019)] or in the Advanced Biological Research System [ABRS, LED lighting; (Paul et al., 2013b)]. Both the EMCS and SIMBOX have a centrifuge, providing the capability for an extremely informative on-orbit 1 x *g* control [and for investigating other fractional *g* environment, e.g., (Correll et al., 2013; Fengler et al., 2015)]. The WS ecotype has been grown in the BRIC, ABRS, Veggie, and in Petri dishes attached to the ISS cabin wall (in both dim diffuse light and in total darkness). Thus, as data from a wide range of experiments has been imported into TOAST, it is important to assess the likely impact of features such as hardware, tissue samples, and seedling age when making comparisons between datasets. For example, differences in atmospheric control and lighting may have important influences on plant responses. Thus, plants grown in the BRIC (darkness, sealed system) might show altered hypoxic response when compared to those in the EMCS (lighting and atmospheric control). Careful attention to the parallel ground controls and, if available, on-orbit centrifuge data are critical to helping understand the extent of such effects. In addition to hardware and growth environment, some specific data features may also impact user analyses. For example, the Ler-0 ecotype was grown in both the EMCS and BRIC but the fact that different microarray technologies [Agilent vs Affymetrix; (Correll et al., 2013; Johnson et al., 2017)] were used in each study needs to be taken into account when making comparisons. This is because results from these different measurement approaches, even when applied to replicate samples have been reported to differ in some cases [e.g., (Del Vescovo et al., 2013)]. Similarly, during the ABRS APEX01 study, Col-0 and WS samples were combined and then separated into roots, stems, and leaves for transcriptional analysis (Paul et al., 2013b). Therefore, when using these datasets allowance for the mixed ecotypes in the sample would need to be made.

In addition to seedlings, cell cultures have been subjected to spaceflight. Thus, Zupanska et al. (2013) compared *Arabidopsis* seedlings and wild type cell cultures grown in the dark within the BRIC. Subsequent spaceflight experiments saw comparisons between wild-type *Arabidopsis* cell cultures and those with mutations in the genes for *ALTERED RESPONSE TO GRAVITY 1* (*ARG1;* a well-studied *Arabidopsis* gene related to gravity sensing) and *HEAT SHOCK FACTOR 2a* [HSF2a; a key heat shock response-related transcriptional regulator; (Zupanska et al., 2017; Zupanska et al., 2019)]. Fengler et al. (2015) also flew *Arabidopsis* and rice cell cultures in the SIMBOX hardware on the Shenzhou-8 spacecraft. Interestingly, despite a large number of differences in the methodologies used in the preparation of the *Arabidopsis* cell cultures between these various experiments (notably culture age and hardware), TOAST analysis identifies three genes that are significantly differentially expressed in all sets of experiments (AT5G48560, *CRY2-INTERACTING BHLH 2*; AT1G73260, *KUNITZ TRYPSIN INHIBITOR 1,* and AT2G15220, a basic secretory protein family member). The sharing of such responses across multiple cell culture spaceflight experiments implies these changes in transcription may be linked to a common element of the spaceflight environment that impacts a physical factor related to spaceflight at a cellular-level. Facilitating such rapid, comparative analyses is a major focus of the TOAST 4.5 architecture.

## Non-Spaceflight Datasets Within TOAST

Many ground-based analyses are relevant to specific aspects of the spaceflight environment. Therefore, several non-spaceflight datasets have been added to the TOAST database to aid with these comparative analyses. Thus, as you move further from the protection of the Earth's magnetic field radiation levels experienced by biological systems increase. Studies using ATH1 microarrays that study radiation effects on plants are therefore also included within TOAST. In these ground-based experiments, wild-type WS seedlings and mutants compromised in DNA repair (*atm-1, atr-1*) were treated with both gamma photons and high-charge, high-energy (HZE) radiation and their transcriptional response monitored (Culligan et al., 2006; Missirian et al., 2014). These studies provide fingerprints of transcriptional response to both increased radiation and increasing levels of DNA damage for comparison to the changes seen in spaceflight datasets.

Likewise, data from *Arabidopsis* cell cultures grown while either experiencing magnetic levitation or growth on random positioning machines (Manzano et al., 2012) are also included in TOAST 4.5. These two techniques have been used to mimic elements of the spaceflight environment such as reduced contact with the substrate and disruption of directional cues normally derived from 1 x *g* on Earth, providing further useful comparisons to spaceflight responses. These gene expression datasets were obtained using the CATMA microarray technology and so some care should be taken when making comparisons to data from experiments using the ATH1 Affymetrix microarray as these two technologies are not identical and e.g., the data from the CATMA arrays was analyzed using the slightly older TAIR9 genome annotation to assign gene IDs (Lamesch et al., 2012).

The BRIC hardware is one of the most widely used plant growth systems for spaceflight and so TOAST also contains a dataset related to growth of plants in the BRIC hardware on Earth to help provide context for analyses in that particular piece of equipment (Basu et al., 2017). Many spaceflight samples are also preserved on orbit in the chemical fixative RNAlater and so TOAST includes a dataset on the effects of RNAlater on *Arabidopsis* seedlings (GLDS-38). In addition, as there are several spaceflight studies that present data on root responses to spaceflight, a root tip transcriptome (Krishnamurthy et al., 2018) and root tissue gene expression mapping (Birnbaum et al., 2003) are also included for comparative analyses.

It is important to note that the ground-based studies incorporated into TOAST are not an exhaustive survey of the publicly available datasets but are intended as an entry point for such comparative analysis. A summary of the non-spaceflight datasets incorporated into TOAST 4.5 is presented in **Supplementary Table 1**.

## TOAST Confirms and Extends Previous Transcriptome Analyses

**Figure 7:**
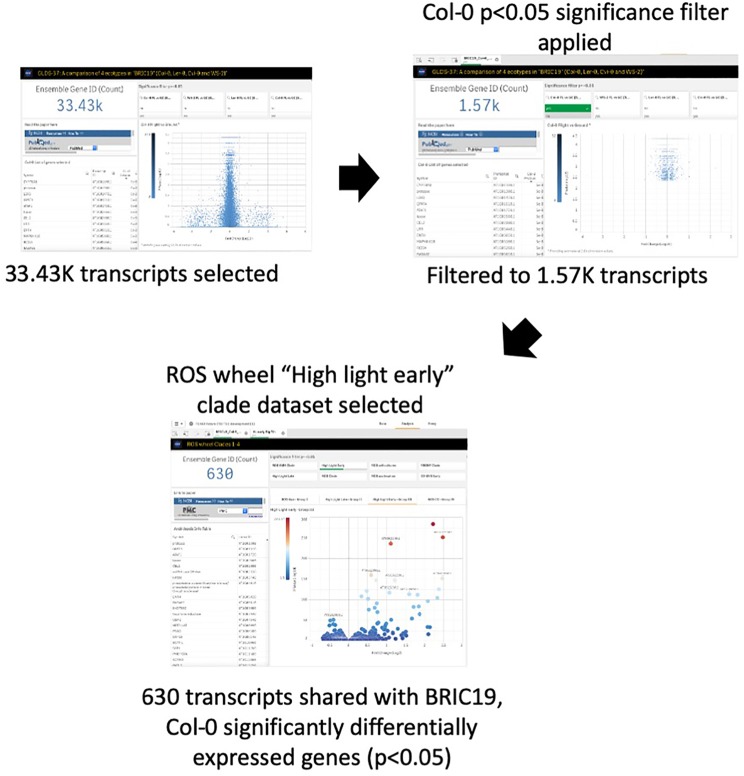
TOAST confirms the “high light early” ROS response from spaceflight data. The “high light early” clade in the ROS wheel analysis represents 8.87K transcripts from a total of 21.33K transcripts detected, or 41.5% of all transcripts.

Oxidative stress has been highlighted as a likely spaceflight-related response in multiple experiments. Therefore, TOAST 4.5 also includes datasets/dashboards for comparative “ROS wheel” analyses. The ROS wheel (Willems et al., 2016) is a meta-analysis of 79 Affymetrix ATH1 microarray studies related to Arabidopsis redox homeostasis experiments. It provides a comprehensive overview of ROS and oxidative stress-related transcriptional signatures, allowing TOAST to filter for ROS-related events within spaceflight datasets. For example, Choi et al. (2019) noted the “high light early” oxidative stress signature from the ROS wheel as a common feature of the responses of *Arabidopsis* in the BRIC-19 spaceflight experiment. “High light early” is one of the groupings (clades) of response defined in the ROS wheel analysis and refers to the common ROS-related transcriptional signature seen in a set of experiments all exposing plants to a high light intensity stress for between 30 min and 2 h. **Figure 7** shows that reanalysis with TOAST confirms this patterning with ~⅓ of the genes significantly altered in spaceflight in Col-0 in the BRIC19 experiment also being significantly modulated in the ROS wheel “high light early” response clade. The power of these comparative approaches is shown using TOAST to perform similar analyses on other spaceflight transcriptomes. Thus, in (Beisel et al., 2019); the APEX3-2 experiment (GLDS-218; using the Veggie hardware and Arabidopsis Col-0 ecotype) TOAST analysis reveals that at 4 days of growth on orbit, 533 of the significantly differentially expressed genes in the root in response to spaceflight were also seen in the “high light early” clade of the ROS wheel. This pattern is reiterated through the time-course of the experiment (day 8, 295 transcripts and at day 11, 29 in the root and 265 in the shoot tissues). Analyses across other spaceflight experiments (**Supplementary Table 4**) shows that such regulation of “high light early” genes is seen in many flight experiments using whole seedlings. Interrogation with the metadata app shows these experiments mostly include plants grown in the dark, suggesting that while the triggering of a “high light early” oxidative stress pathway may be a common response of plant biology to some feature of the spaceflight environment, this is unlikely to be due to high light levels.

## Cross-Species Analyses Using TOAST

TOAST 4.5 also allows for seamless cross-species comparisons that offer the possibility to reveal fundamental elements of plant biology response to spaceflight. For example, when we used TOAST to compare the significantly differentially expressed genes in rice cell cultures grown on the Shenzhou 8 spacecraft with the *Arabidopsis* cell cultures from the same flight, 483 orthologous loci were identified (filtering on P-value <0.05, Q-value < 0.05 and for genes mapping to unique Ensembl gene Identifiers; **Supplementary Table 5**). The expression of, for example, genes encoding receptor-like kinases thought to be involved in response to pathogens were altered in both species, indicating that spaceflight-induced changes in the response system to biotic stress might be a conserved plant spaceflight response. Importantly, these samples were grown under sterile conditions on orbit, suggesting these responses were triggered without pathogen stimulus. In addition, both cell cultures showed changes in the expression of genes related to cell wall structure, a theme already highlighted in several reports on *Arabidopsis* seedlings grown in spaceflight [e.g., (Choi et al., 2014; Kwon et al., 2015; Johnson et al., 2017)] and readily discernable as a transcriptional pattern from TOAST analyses of these same spaceflight samples. Comparison between the datasets from these cell culture samples grown under microgravity with those in the 1 x *g* on-orbit centrifuge control module within the SIMBOX hardware of this experiment showed 111 of the genes that were significantly differentially expressed in spaceflight vs ground controls in both *Arabidopsis* and rice cultures were also differentially expressed in the flight vs 1 x *g* centrifuge (**Supplementary Table 5**). That is, these genes were most likely not responding to the microgravity component of the spaceflight environment (which is nullified by centrifugation). Thus, some other feature(s) of spaceflight, such as increased background radiation or the development of microgravity-induced hypoxia [e.g., (Choi et al., 2019)] may be affecting this particular response.

## TOAST: Survey of Spaceflight Responsive Genes Implies Alterations in Mitochondrial Function

**Figure 8:**
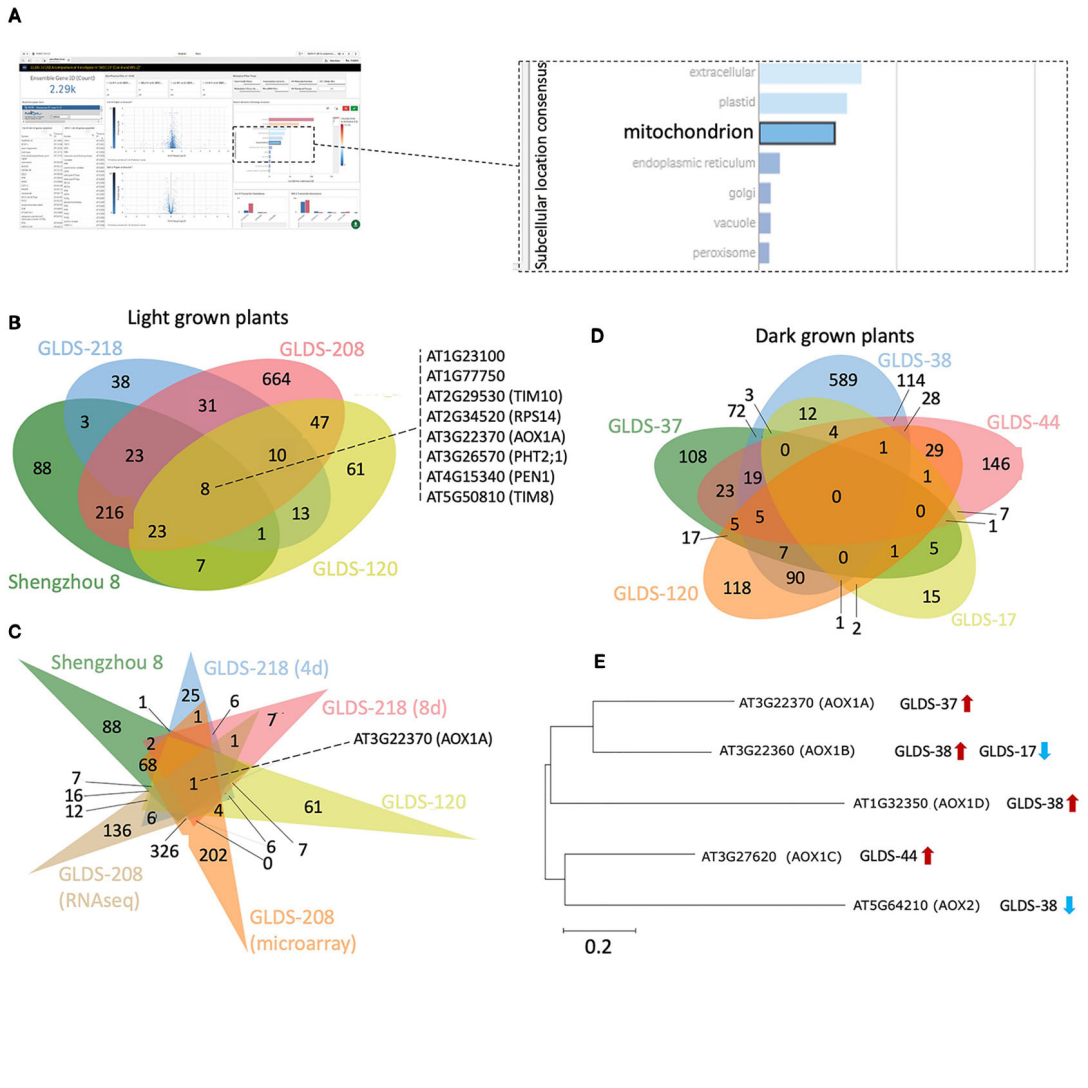
Analysis of mitochondrion-related genes altered by spaceflight. **(A)** Screenshot depicting an example of a user's interaction with the TOAST graphical user interface to define mitochondrion-related transcripts. **(B)** Using TOAST for iterative filtering of differentially expressed genes across multiple spaceflight studies where plants were light grown. **(C)** More extensive analysis of the studies in **(B)** using differentiation within the individual datasets for different analytical approaches (microarray vs RNAseq) and for different analysis periods (4 days vs 8 days). **(D)** Similar analysis but for dark grown plant samples. **(E)** The effects of spaceflight on the alternative oxidase gene family in dark grown samples. Maximum likelihood tree of AOX gene family generated using ClustalW alignment with Mega-X software (www.megasoftware.net). Venn diagrams plotted using jvenn (Bardou et al., 2014).

Manual inspection of the subcellular locations presented in the TOAST interface suggested to us a potentially common element: mitochondria-related transcripts appeared to often be significantly altered in spaceflight samples. Therefore, to more closely identify possibly conserved spaceflight-related changes to mitochondrial function, 2,290 genes annotated as belonging to the “mitochondrion” were selected using the “GO subcellular location” tool embedded in TOAST's graphical user interface as shown in **Figure 8A**. Using this filter, significantly differentially expressed genes (P < 0.05) were acquired from the analyses of *Arabidopsis* Col-0 plants grown in space in either light or dark conditions. In total, 1,233 unique differentially expressed mitochondrial genes were identified from the following light-grown experiments: root tips in CARA (GLDS-120), roots from both four and 8 day old seedlings cultivated in APEX-03's Veggie growth system (GLDS-218), the elongation zones of seedlings (GLDS-208), and undifferentiated cell cultures flown in Shenzhou 8's SIMBOX plant growth hardware (Fengler et al., 2015). **Figure 8B** shows that of these 1,233 differentially expressed transcripts, 382 were identified as being shared between at least two datasets, with eight genes being shared across all four experiments (**Supplementary Table 5**). When further comparisons were made using different sample times or assay types as a further distinction within these data (**Figure 8C**), only one gene, alternative Oxidase 1A (AOX1A) was found to be common amongst the significantly differentially expressed genes in all conditions of the four selected experiments. These results suggest analysis of plant mitochondrial functioning during spaceflight may be a fruitful area of research. Indeed, Sugimoto et al. (2014) previously identified an alternative oxidase in mizuna grown on the ISS in the Lada growth chamber (i.e., in the light) as showing 9.2-fold induction during spaceflight. While the majority of the selected experiments report induction of *AOX1A*, instances of repression were also identified in roots extracted from four day and 8 day old seedlings grown in APEX-03's Veggie growth system.

Furthermore, *AOX1A* is significantly induced in both the SIMBOX “Flight Static” vs “Ground Static” analyses (i.e. samples grown in microgravity compared to ground controls), and in the ‘Flight Centrifuge' vs “Ground Static” comparisons (i.e., plants grown at 1 x *g* on orbit vs ground controls not in a centrifuge). However, no significant difference of expression is observed when comparing the “Flight Static” vs “Flight Centrifuge” environments. These results highlight the power of being able to make comparisons to an on-board 1 x *g* control. The data suggest that the induction of *AOX1A* in light-grown undifferentiated cells is likely not a microgravity-driven event but reflects some other aspect of the spaceflight environment, such as increased radiation exposure, possible development of hypoxia or altered fluid dynamics.

The datasets chosen for the TOAST analysis above that highlight *AOX1A* originate from experiments with samples grown under light. To explore whether the light environment might be playing a role in this suite of responses, several “dark-grown” spaceflight studies of the Col-0 ecotype were also selected using the TOAST metadata app: etiolated seedlings and undifferentiated cell cultures grown aboard BRIC19 (GLDS-37), BRIC20 (GLDS-38), BRIC16 (GLDS-17, GLDS-44), and etiolated root tips extracted from the CARA experiment (GLDS-120). Comparisons between these datasets revealed no commonly regulated genes (**Figure 8D**) and *AOX1A* was only significantly differentially expressed in the BRIC19 study in this analysis. Therefore, we examined the spaceflight-related transcriptional responses in the other members of the *AOX* gene family (**Figure 8E**). Indeed, other alternative oxidases are differentially expressed in these other “dark-grown” experiments, with each *AOX* gene being differentially expressed in at least one selected “dark-grown” study (**Figure 8E**). Given these altered expression patterns of members of the *AOX* family in multiple experiments, these results suggest that the regulation of alternative oxidases in response to spaceflight-associated stressors would be a strong candidate for future research studies.

Thus, this analysis in TOAST suggests a potentially widespread alteration in mitochondrial function in plants experiencing spaceflight, but many questions arise from these observations. Is an alternative oxidase pathway being triggered by spaceflight stress? Could mitochondrial dysfunction be a significant element in the oxidative stress responses seen in plant spaceflight data, as suggested e.g., for mammalian ocular tissues (Mao et al., 2013) or osteoblasts experiencing microgravity (Michaletti et al., 2017)? This kind of comparative data mining highlights the possibilities for hypothesis generation supported by the TOAST environment.

However, here it is important to note some of the limitations inherent in these kinds of analyses. For example, hypoxia is thought to be imposed during spaceflight by local oxygen consumption and associated depletion around metabolically active tissues. The reduced convective gas mixing inherent in microgravity (Porterfield, 2002) then lowers oxygen resupply leading to development of a depletion zone around these tissues. Such hypoxic stress would be an obvious potential modulator of mitochondrial function. Yet, hypoxic signatures do not readily emerge from GO analysis of the transcript profiles of the plant spaceflight datasets, yet hypoxia is a term that GO enrichment analyses can highlight. Thus, one possibility is that another, yet to be defined, physical element(s) of the spaceflight environment may act to drive these changes in mitochondrial function. However, the formation of hypoxic environments due to microgravity is likely to be very different from how hypoxia either develops naturally on Earth, or can be experimentally imposed in ground-based experimentation. For example, the steep local oxygen depletion zones that form in microgravity are more likely to be disrupted by convective gas mixing on Earth. This observation highlights one of the important caveats of relying strongly on GO analyses to understand spaceflight data. Gene ontology analyses match patterns of gene expression to those seen under particular conditions on Earth. Therefore, how well treatments on Earth mimic conditions developing during spaceflight may affect the sensitivity of such GO analyses for defining these spaceflight responses.

Similarly, it is important to ask how much batch effects might be superimposed on any particular analysis (Leek et al., 2010). Batch effects are where measurements are impacted by a non-biological treatment related factor that systematically changes the measurement. For example, for RNAseq, a batch effect might be differences in patterns of gene expression related to the day a particular set of samples was processed for sequencing rather than the biological treatment of the samples. For microarray analyses it could be differences imposed by different batches of microarray being used for different sets of samples. Batch effects can be complex to resolve but statistical approaches such as surrogate variable analysis (Leek and Storey, 2007; Leek et al., 2010) can be used on a case-by-case basis by the researcher to estimate the sensitivity of a particular dataset's analysis to these kinds of effects and so help build a case for the robustness of the analysis.

## Conclusions

As the volume of spaceflight omics-level data increases, its power will lie in researchers' ability to mine both within and across multiple datasets. Such comparisons will provide an important source of hypotheses to then be experimentally tested. TOAST provides a data-rich environment with which to explore the commonalities and differences in the responses of plants to spaceflight and spaceflight-related environments in an accessible and intuitive format. The TOAST database has been released as a publicly available, web-based environment (https://astrobiology.botany.wisc.edu/astrobotany-toast) along with an online tutorial at https://astrobiology.botany.wisc.edu/astrobotany-toast/tutorial-metadata. At present, TOAST provides a tool to aid the plant biology community. However, the underlying TOAST architecture is biological kingdom agnostic; through use of orthologous matrix mapping, we are working to extend TOAST to facilitate similar data exploration across the wealth of biological systems that are being analyzed in spaceflight.

## Data Availability Statement

The datasets analyzed for this study can be found in the GeneLab data repository (https://genelab-data.ndc.nasa.gov/genelab/projects/) and the Gene Expression Omnibus (https://www.ncbi.nlm.nih.gov/geo/).

## Author Contributions

RB developed the database. RB and KR developed the user interface and tutorial. RB, JL, and SG analyzed data and wrote the manuscript.

## Funding

This research was funded by NASA grants NNX13AM50G, NNX14AT25G, NNX17AD52G, 80NSSC18K0126, 80NSSC18K0132. The Qlik software used in this work is provided under a free-to-use educational license from QlikTech International.

## Conflict of Interest

The authors declare that the research was conducted in the absence of any commercial or financial relationships that could be construed as a potential conflict of interest.

## Appendix

**Appendix:**

Acronym/term	Name	Definition	Reference
ABRS	Advanced Biological Research System	NASA on-orbit growth facility that provided LED lighting and sample photography	(Paul et al., 2012; Paul et al., 2013b)
Affymetrix microarray	–	Microarray to monitor patterns of gene expression produced by Affymetrix Inc.	–
AGI	Arabidopsis Gene Initiative	Consortium of researchers studying the genome of *Arabidopsis thaliana*	(Kaul et al., 2000)
AGRIS AtTFD	AGRIS Arabidopsis Transcription Factor database	A searchable database of ~1770 *Arabidopsis thaliana* transcription factors grouped into families by conserved domain structures. Maintained by The Arabidopsis Gene Regulatory Information Server (AGRIS).	(Palaniswamy et al., 2006)
ATTED II	*Arabidopsis thaliana* trans-factor and cis-element prediction database	A database cataloging plant gene co-expression data	(Obayashi et al., 2018)
BAM	Binary compressed sequence Alignment Map	A file containing information on the alignment of each read from a DNA sequencing machine to the genome of a target organism	–
BRIC	Biological Research in Canister	Spaceflight hardware allowing for plant growth on orbit. Samples are sealed prior to launch. Lighting provided only in the BRIC-LED version	
CATdb	–	A repository of transcriptome data for *Arabidopsis thaliana* produced by the Complete Arabidopsis Transcriptome Micro Array (CATMA) platform	(Gagnot et al., 2008)
CATMA microarray	Complete Arabidopsis Transcript MicroArray	Microarray to monitor patterns of gene expression using technology developed by the European CATMA initiative.	(Sclep et al., 2007; Gagnot et al., 2008)
CPM	counts per million reads mapped	In RNAseq: the counts of number of reads per gene scaled to the number of fragments sequenced. Unlike FPKM (see below), this value is not normalized for the effects of gene length or amount of sequencing on count number per gene.	–
CyVerse	–	A cloud computing infrastructure supported through the National Science Foundation's Directorate of Biological Sciences.	(Goff et al., 2011; Merchant et al., 2016)
D3 JavaScript	–	A library of routines for the Javascript programming language that enables interactive data visualizations within a web browser.	–
DESeq	–	An analysis tool for calculating differential gene expression.	(Anders and Huber, 2010; Love et al., 2014)
EdgeR	Empirical analysis of Digital Gene Expression in R	An analysis tool calculating differential gene expression.	(Robinson et al., 2009; Love et al., 2014)
eFP-Seq Browser	–	An RNA-seq data exploration and visualization tool.	(Sullivan et al., 2019)
EMBL EBI Expression atlas	–	A database of patterns of gene expression under different conditions. Maintained by The European Molecular Biology Laboratory's (EMBL) European Bioinformatics Institute (EBI).	(Papatheodorou et al., 2018)
EMCS	European Modular Cultivation System	On-orbit growth hardware developed by the European Space Agency. Provides an on-board centrifuge, video and lighting, temperature and atmospheric control.	(Correll et al., 2013; Mazars et al., 2014; Vandenbrink et al., 2019)
Ensembl	–	A database of genome-related information maintained by the European Bioinformatics Institute and the Wellcome Trust Sanger Institute,	(Kersey et al., 2018; Zerbino et al., 2018)
Entrez	–	The US National Center for Biotechnology Information (NCBI)'s database for gene-specific information	(Maglott et al., 2011)
ePlant	–	A portal that provides access to multiple web services to download genome-level data on plant genes.	(Waese et al., 2017)
Expression Angler	–	A tool that finds other genes with similar expression patterns to a gene of interest.	(Austin et al., 2016)
FASTQ file	–	File containing the nucleotide sequences identified by next generation nucleotide sequencing machines	–
FPKM	Fragments Per Kilobase of transcript per Million mapped reads	An estimation of gene expression based on RNA-sequencing data that is normalizing for gene length and the amount of sequencing (longer and more heavily sequenced genes will naturally produce more reads independent of their expression level).	–
Gene Symbol	–	Commonly used gene name such as AOX1A to denote the Arabidopsis gene *ALTERNATIVE OXIDASE 1A*	–
GeneLab	–	A repository for spaceflight-related ‘omics-level data administered by the US National Aeronautics and Space Administration (NASA).	–
Genemania	Gene Multiple Association Network Integration Algorithm	A tool that generates a single functional interaction network for a gene of interest drawing on multiple data sources.	(Franz et al., 2018)
Genevisble	–	A search portal to curated expression data from the GENEVESTIGATOR database	(Hruz et al., 2008)
GEO	Gene Expression Omnibus	A functional genomics data repository administered by US National Center for Biotechnology Information (NCBI).	–
GLDS	GeneLab Dataset	Unique identifier of a dataset (usually microarray, RNAseq or proteomics data) deposited in NASA's GeneLab data repository	–
GO	Gene Ontology	Descriptive terms drawn from a standard set that classify genes dependent on their relationships to biological processes or functions or subcellular locales.	(Ashburner et al., 2000; Carbon et al., 2019)
GO Enrichment analysis	Gene Ontology Enrichment analysis	Statistical analysis of dataset as to whether there is an over-representation of genes associated with a particular biological process or function, or cellular locale relative to that expected from a random selection of the same number of genes.	(Ashburner et al., 2000; Carbon et al., 2019)
HZE	–	High-charge, high-energy radiation.	–
iDEP	integrated Differential Expression and Pathway analysis	Software package for the R programming language designed to process genetic data.	(Ge et al., 2018)
KEGG	Kyoto Encyclopedia of Genes and Genomes	A widely used database that categorizes genes into the cellular pathways in which they are involved.	(Kanehisa et al., 2017)
Metadata	–	Additional data about parameters and conditions that adds to the description of each experiment and provides context for interpreting results.	–
microRNA annotation TAIR10	–	A database of microRNAs predicted in the genome of *Arabidopsis thaliana* from the TAIR 10 genome annotation.	(Lamesch et al., 2012)
NCBI	The National Center for Biotechnology Information	Part of the National Library of Medicine that is run by the US National Institutes of Health. This unit maintains a series of databases relevant to biological research	–
NCBI PubMed	–	Online aggregator of scientific publications curated by NCBI	–
OM	Orthologous Matrix	A table linking gene identifiers in one species to orthologous genes in a different species	
Ortholog	–	Related genes between species that originated from a common ancestral gene prior to speciation	–
P-value vs Q-value	–	In transcriptomics: P-value is the statistical significance that a gene is differentially expressed when comparing between treatments; Q-value is an adjusted P-value, taking in to account the cumulative effect of making multiple comparisons (tests of significance) within a dataset, such as across many genes.	
Promomer	–	A tool for identifying promoter elements	(Toufighi et al., 2005)
Qlik	–	Database management software	–
R	–	Programming language widely used in the statistical analysis of scientific data.	–
R-Shiny	–	An R software package that allows for easy development of interactive web-based applications.	–
R-studio	–	Commercially produced software that aids with the development of programs using R.	–
Reactome	–	A curated and peer-reviewed molecular pathway database	(Fabregat et al., 2018)
RMA	Robust Multi-array Average	An algorithm used to normalize microarray data between multiple microarray chips	(Irizarry et al., 2003)
RNA-seq	–	High-throughput sequencing of RNA.	–
ROS-wheel	–	A meta-analysis of many publicly available microarray experiments related to responses to reactive oxygen species (ROS) and oxidative stress.	(Willems et al., 2016)
SIMBOX	Science In Microgravity BOX	An on-orbit experiment facility developed by the German Aerospace Center's (DLR) Space Administration. Contains an internal centrifuge and lighting and temperature control.	(Preu and Braun, 2014)
STRING	–	A database and web tool for visualizing protein:protein interaction networks.	(Szklarczyk et al., 2019)
SUBA4	The SUBcellular location database for Arabidopsis	Database of predicted subcellular locations for a given gene product.	(Hooper et al., 2017)
TAIR	The Arabidopsis Information Resource	A database of genetic and molecular biology data focused on *Arabidopsis thaliana*	(Berardini et al., 2015)
TAIR9/TAIR10	The Arabidopsis Genome Annotation Version 9 or 10	Annotated versions of the sequenced Arabidopsis genome produced by TAIR. Each successive version has used newer information to improve the annotation of the entire genome.	–
Thalemine	–	A data warehouse aggregating many genomic tools and datasets for *Arabidopsis thaliana*.	(Krishnakumar et al., 2016)
TOAST	Test Of Arabidopsis Space Transcriptome database	A relational database that compares plant biology, spaceflight-related omics datasets and their associated metadata.	–
Veggie	–	NASA's Vegetable Production System; an ISS-based growth hardware providing LED lighting.	(Massa et al., 2017)
Volcano plot		A scatter plot of data. For the microarray and RNAseq data in TOAST the volcano plot presents fold-change per gene ID plotted versus statistical significance for each data point.	
