# Supplementary material for: Test of Arabidopsis Space Transcriptome: A Discovery Environment to Explore Multiple Plant Biology Spaceflight Experiments
Source: Front Plant Sci. 2020 Mar 4;11:147. doi: 10.3389/fpls.2020.00147 (PMC7076552; doi:10.3389/fpls.2020.00147)
Supplement: Supplementary file 1 [file DataSheet_1.docx]

Supplementary Material

**Supplementary Table 1. Plant spaceflight datasets and ground-based datasets related to spaceflight factors.** A small selection of meta-data associated with these studies is also included to indicate the breadth of the data available. GeneLab, GeneLab data repository (https://genelab-data.ndc.nasa.gov/); GEO, Gene Expression Omnibus (https://www.ncbi.nlm.nih.gov/geo/); Col-0, Columbia ecotype of *Arabidopsis thaliana*; Ws, Wassilewskija ecotype; Ler, Landsberg ecotype; Cvi, Cape Verdi islands ecotype; mutants of Arabidopsis: *phyD*, Phytochrome D; *arg*, Altered Response to Gravity; *act2*, Actin 2; *ATM,* Ataxia-telangiectasia mutated*; atr,* ATM and Rad 3-related*; xpf,* xeroderma pigementosa F endonuclease; RPM, random positioning machine; HZE, High atomic weight and high energy; ROS Meta, meta analysis of experiments related to reactive oxygen species response; BRIC, Biological Research in Canister.

| **Identifier** | **Reference** | | **Genotype or ecotype**  (*Arabidopsis* *thaliana* unless noted) | | **Notes** | | | **Assay Method** | | |  |  |  |
| --- | --- | --- | --- | --- | --- | --- | --- | --- | --- | --- | --- | --- | --- |
| **Spaceflight** | | | | | | | | | | | |  |  |
| GLDS-7 | | (Paul et al., 2013) | | WS & Col | | Flight vs Ground | | | Microarray | | | |  |
| GLDS-16 | | (Ferl et al., 2015) | | WS | | “ | | | Proteomics | | | |  |
| GLDS-17 | | (Paul et al., 2012) | | Col | | “ | | | Microarray | | | |  |
| GLDS-37 | | (Choi et al., 2019) | | Col, WS, Ler, Cvi | | “ | | | RNAseq | | | |  |
| GLDS-38 | | NA | | Col | | “ | | | RNAseq | | | |  |
| GLDS-44 | | (Kwon et al., 2015) | | Col, *act2-3* | | “ | | | Microarray | | | |  |
| GLDS-57 | | (Salmi and Roux, 2008) | | *Ceratopteris reinhardii* | | “ | | | Microarray | | | |  |
| GLDS-59 | | (Sugimoto et al., 2014) | | *Brassica napa* | | “ | | | RNAseq | | | |  |
| GLDS-120 | | (Paul et al., 2017) | | *phyD*, WS, Col | | “ | | | RNAseq | | | |  |
| GLDS-121 | | (Johnson et al., 2017) | | Ler | | “ | | | Microarray | | | |  |
| GLDS-147 | | (Zupanska et al., 2017) | | Col, *arg1* | | “ | | | Microarray | | | |  |
| GLDS-205 | | (Zupanska et al., 2019) | | Col, *hsfa2* | | “ | | | Microarray | | | |  |
| GLDS-213 | | (Fengler et al., 2015) | | Col | | “ | | | Microarray | | | |  |
| GLDS-217 | | (Zhou et al., 2019) | | Col & WS | | “ | | | RNAseq | | | |  |
| GLDS-218 | | (Beisel et al., 2019) | | Col | | “ | | | RNAseq | | | |  |
| GLDS-251 | | (Vandenbrink et al., 2019; Herranz et al., 2019) | | Col | | “ | | | RNAseq | | | |  |
| NA | | (Correll et al., 2013) | | Ler | | “ | | | Microarray | | | |  |
| NA | | (Jin et al., 2015) | | *Oryza sativa* | | “ | | | Microarray | | | |  |
| **Spaceflight-related, ground-based studies** | | | | | | | | | | | |  |  |
| GLDS-38 | | (Kruse et al., 2017) | | Col | | | RNA later treatment | | | RNAseq & Proteomics | | | |
| GEO: GSE29787 | | (Manzano et al., 2012) | | Col | | | RPM, magnetic levitation | | | Microarray | | | |
| NA | | (Culligan et al., 2006) | | WS, *atm, atr, xpf‐2* | | | 10Gy radiation | | | Microarray | | | |
| GLDS-46 / GEO: GSE61484 | | (Missirian et al., 2014) | | Col and *atm1-1* | | | HZE & Gamma radiation | | | Microarray | | | |
| GLDS-22 | | (Visscher et al., 2010) | | Col & *cax1-1* | | | MgCl_2_ salts | | | Microarray | | | |
| NA | | (Willems et al., 2016) | | Col, Ler, WS | | | ROS Meta analysis | | | Microarray | | | |
| GEO: GSE5749 | | (Birnbaum et al., 2003) | | Col | | | Root tissue map | | | Microarray | | | |
| NA | | (Basu et al., 2017) | | Col | | | BRIC hardware effect | | | RNAseq, proteomics | | | |
| GLDS-208 | | (Krishnamurthy et al., 2018) | | Col | | | Root tip transcriptome | | | Microarray & RNAseq | | | |
| GLDS-144 | | (Kamal et al., 2019) | | Col | | | RPM, cell cycle analysis | | | Microarray | | | |
| GLDS-45 | | NA | | Col | | | Clinostat responses | | | Microarray | | | |
| GLDS-8 | | (Manzano et al., 2012) | | Ler | | | Magnetic levitation | | | Microarray | | | |

**Supplementary Table 2:** Tools included in the TOAST database graphical user interface.

| **Database / tool linked** | **Use** | **Reference** |
| --- | --- | --- |
| SUBA4 | Subcellular consensus | (Hooper et al., 2017) |
| ATTED | Symbol & ortholog network | (Obayashi et al., 2018) |
| ePlant | Gene viewer | (Waese et al., 2017) |
| GeneSlider | Gene promoter viewer | (Waese et al., 2017) |
| Thalemine | Gene & network viewer | (Krishnakumar et al., 2016) |
| Expression Angler | Co-expressed genes | (Austin et al., 2016) |
| eFP-Seq Browser | Aggregated RNAseq search | (Sullivan et al., 2019) |
| Promomer | Network Promoter Viewer | (Toufighi et al., 2005) |
| STRING | Network viewer | (Szklarczyk et al., 2019) |
| Genemania | Network viewer | (Franz et al., 2018) |
| Arabidopsis interaction viewer | Arabidopsis Network Viewer | (Geisler-Lee et al., 2007) |
| Rice interaction viewer | Rice Network Viewer | (Ho et al., 2012) |
| Genevisble | RNAseq visualization | (Hruz et al., 2008) |
| CATdb | Access processed microarrays | (Gagnot et al., 2008) |
| AGRIS TF database | Transcription factor filter | (Palaniswamy et al., 2006) |
| microRNA annotation TAIR10 | Gene set filter | (Lamesch et al., 2012) |
| Gene Families TAIR10 selection | Gene set filter | (Lamesch et al., 2012) |
| EBI Expression atlas | Access processed microarrays | (Papatheodorou et al., 2018) |
| Ensemble Plants | Access to plant genome annotation | (Kersey et al., 2018) |
| Reactome | Access to plant reaction data | (Fabregat et al., 2018) |

**Supplementary Table 3. Experimental factors related to plant biology studies within the TOAST Metadata app.** Manual curation of the metadata associated with the spaceflight-related experiments in TOAST 4.5.

See attached Excel file

**Supplementary Table 4. Significantly differentially expressed transcripts in spaceflight vs those also seen in the “high light early” clade of the ROS wheel analysis.** DET, differentially expressed transcripts. The “high light early” clade in the ROS wheel analysis consists of 8.89K genes identified by meta analysis of publicly available microarray data (Willems et al., 2016).

| Dataset | Sample | Light/dark  grown | DET  P<0.05 | “High Light Early” clade members |
| --- | --- | --- | --- | --- |
| GLDS-7 | Seedling, Ws | Light | 2.96K | 1.25K |
| GLDS-17 | Seedling, Col | Dark | 1.34K | 719 |
| GLDS-37 | Seedling, Ler | Dark | 451 | 179 |
| GLDS-37 | Seedling, Cvi | Dark | 2.27K | 1.02K |
| GLDS-37 | Seedling, Ws | Dark | 903 | 380 |
| GLDS-44 | Seedling, Col | Dark | 6.32K | 2.87K |
| GLDS-121 | Seedling, Ler | Dark | 239 | 144 |

**Supplementary Table 5.** Orthologous loci responding to spaceflight in Arabidopsis and rice cell cultures grown during spaceflight. Orthologs defined using Orthologous Matrix (Altenhoff et al., 2018) mapping within TOAST 4.5. Table shows Arabidopsis gene symbol, Arabidopsis gene ID, orthologous rice gene ID, % identity between Arabidopsis and rice ortholog and adjusted P-value (Q-value) for the Arabidopsis data and the orthologous rice gene’s data. Table 5.1 compares genes significantly differentially expressed (P<0.05) in spaceflight vs ground controls in Arabidopsis and rice cell cultures described in Fengler et al. (2015). Table 5.2 filters these gene lists for Q<0.05 for both Arabidopsis and rice datasets. Table 5.3 shows genes significantly differentially expressed (P<0.05) in Arabidopsis and rice cultures in flight (microgravity) samples versus samples in the 1 x *g* on-board centrifuge. Table 5.4 filters the data in Table 5.3 on Q-value <0.05 and then compares the genes that are significantly differentially expressed (P<0.05, Q<0.05) in both Arabidopsis and rice cell cultures in the flight vs ground control comparison with those significantly differentially expressed (P<0.05, Q<0.05) in both Arabidopsis and rice cell cultures in the flight vs centrifuge dataset.

See attached Excel Spreadsheet

**Supplementary Table References**

Altenhoff, A. M., Glover, N. M., Train, C. M., Kaleb, K., Warwick Vesztrocy, A., Dylus, D., et al. (2018). The OMA orthology database in 2018: Retrieving evolutionary relationships among all domains of life through richer web and programmatic interfaces. *Nucleic Acids Res.* 46, D477–D485. doi:10.1093/nar/gkx1019.

Austin, R. S., Hiu, S., Waese, J., Ierullo, M., Pasha, A., Wang, T. T., et al. (2016). New BAR tools for mining expression data and exploring Cis-elements in *Arabidopsis thaliana*. *Plant J.* 88, 490–504. doi:10.1111/tpj.13261.

Basu, P., Kruse, C. P. S., Luesse, D. R., and Wyatt, S. E. (2017). Growth in spaceflight hardware results in alterations to the transcriptome and proteome. *Life Sci. Sp. Res.* 15, 88–96. doi:10.1016/j.lssr.2017.09.001.

Beisel, N. S., Noble, J., Barbazuk, W. B., Paul, A. L., and Ferl, R. J. (2019). Spaceflight-induced alternative splicing during seedling development in *Arabidopsis thaliana*. *npj Microgravity* 5, 9. doi:10.1038/s41526-019-0070-7.

Birnbaum, K., Shasha, D. E., Wang, J. Y., Jung, J. W., Lambert, G. M., Galbraith, D. W., et al. (2003). A gene expression map of the Arabidopsis root. *Science (80-. ).* 302, 1956–1960. doi:10.1126/science.1090022.

Choi, W. G., Barker, R. J., Kim, S. H., Swanson, S. J., and Gilroy, S. (2019). Variation in the transcriptome of different ecotypes of *Arabidopsis thaliana* reveals signatures of oxidative stress in plant responses to spaceflight. *Am. J. Bot.* 106, 123–136. doi:10.1002/ajb2.1223.

Correll, M. J., Pyle, T. P., Millar, K. D. L., Sun, Y., Yao, J., Edelmann, R. E., et al. (2013). Transcriptome analyses of *Arabidopsis thaliana* seedlings grown in space: Implications for gravity-responsive genes. *Planta*. doi:10.1007/s00425-013-1909-x.

Culligan, K. M., Robertson, C. E., Foreman, J., Doerner, P., and Britt, A. B. (2006). ATR and ATM play both distinct and additive roles in response to ionizing radiation. *Plant J.* 48, 947–961. doi:10.1111/j.1365-313X.2006.02931.x.

Fabregat, A., Jupe, S., Matthews, L., Sidiropoulos, K., Gillespie, M., Garapati, P., et al. (2018). The Reactome Pathway Knowledgebase. *Nucleic Acids Res.* 46, D649-655. doi:10.1093/nar/gkx1132.

Fengler, S., Spirer, I., Neef, M., Ecke, M., Nieselt, K., and Hampp, R. (2015). A whole-genome microarray study of *Arabidopsis thaliana* semisolid callus cultures exposed to microgravity and nonmicrogravity related spaceflight conditions for 5 days on board of Shenzhou 8. *Biomed Res. Int.* 2015, 547495. doi:10.1155/2015/547495.

Ferl, R. J., Koh, J., Denison, F., and Paul, A. L. (2015). Spaceflight induces specific alterations in the proteomes of Arabidopsis. *Astrobiology* 15, 32–56. doi:10.1089/ast.2014.1210.

Franz, M., Rodriguez, H., Lopes, C., Zuberi, K., Montojo, J., Bader, G. D., et al. (2018). GeneMANIA update 2018. *Nucleic Acids Res.* 46, W60–W64. doi:10.1093/nar/gky311.

Gagnot, S., Tamby, J. P., Martin-Magniette, M. L., Bitton, F., Taconnat, L., Balzergue, S., et al. (2008). CATdb: A public access to Arabidopsis transcriptome data from the URGV-CATMA platform. *Nucleic Acids Res.* 36, D986–D900. doi:10.1093/nar/gkm757.

Geisler-Lee, J., O’Toole, N., Ammar, R., Provart, N. J., Millar, A. H., and Geisler, M. (2007). A predicted interactome for Arabidopsis. *Plant Physiol.* 145, 317–329. doi:10.1104/pp.107.103465.

Herranz, R., Vandenbrink, J. P., Villacampa, A., Manzano, A., Poehlman, William, L., Feltus, Frank, A., et al. (2019). RNAseq analysis of the response of *Arabidopsis thaliana* to fractional gravity under blue-light stimulation during spaceflight. *Front. Plant Sci.* 26. doi:10.3389/fpls.2019.01529.

Ho, C. L., Wu, Y., Shen, H. bin, Provart, N. J., and Geisler, M. (2012). A predicted protein interactome for rice. *Rice* 5, 15. doi:10.1186/1939-8433-5-15.

Hooper, C. M., Castleden, I. R., Tanz, S. K., Aryamanesh, N., and Millar, A. H. (2017). SUBA4: The interactive data analysis centre for Arabidopsis subcellular protein locations. *Nucleic Acids Res.* 45, D1064-1074. doi:10.1093/nar/gkw1041.

Hruz, T., Laule, O., Szabo, G., Wessendorp, F., Bleuler, S., Oertle, L., et al. (2008). Genevestigator V3: A reference expression database for the meta-analysis of transcriptomes. *Adv. Bioinformatics* 2008, 420747. doi:10.1155/2008/420747.

Jin, J., Chen, H., and Cai, W. (2015). Transcriptome analysis of *Oryza sativa* calli under microgravity. *Microgravity Sci. Technol.* 27, 437–453. doi:10.1007/s12217-015-9432-2.

Johnson, C. M., Subramanian, A., Pattathil, S., Correll, M. J., and Kiss, J. Z. (2017). Comparative transcriptomics indicate changes in cell wall organization and stress response in seedlings during spaceflight. *Am. J. Bot.* 104, 1219–1231. doi:10.3732/ajb.1700079.

Kamal, K. Y., van Loon, J. J. W. A., Medina, F. J., and Herranz, R. (2019). Differential transcriptional profile through cell cycle progression in Arabidopsis cultures under simulated microgravity. *Genomics* S0888-7543, 30536. doi:10.1016/j.ygeno.2019.01.007.

Kersey, P. J., Allen, J. E., Allot, A., Barba, M., Boddu, S., Bolt, B. J., et al. (2018). Ensembl Genomes 2018: An integrated omics infrastructure for non-vertebrate species. *Nucleic Acids Res.* 46, D802–D808. doi:10.1093/nar/gkx1011.

Krishnakumar, V., Contrino, S., Cheng, C.-Y., Belyaeva, I., Ferlanti, E. S., Miller, J. R., et al. (2016). ThaleMine: A warehouse for Arabidopsis data integration and discovery. *Plant Cell Physiol.* 58, 200. doi:10.1093/pcp/pcw200.

Krishnamurthy, A., Ferl, R. J., and Paul, A. L. (2018). Comparing RNA-Seq and microarray gene expression data in two zones of the Arabidopsis root apex relevant to spaceflight. *Appl. Plant Sci.* 6, e01197. doi:10.1002/aps3.1197.

Kruse, C. P. S., Basu, P., Luesse, D. R., and Wyatt, S. E. (2017). Transcriptome and proteome responses in RNAlater preserved tissue of *Arabidopsis thaliana*. *PLoS One* 12, e0175943. doi:10.1371/journal.pone.0175943.

Kwon, T., Alan Sparks, J., Nakashima, J., Allen, S. N., Tang, Y., and Blancaflor, E. B. (2015). Transcriptional response of Arabidopsis seedlings during spaceflight reveals peroxidase and cell wall remodeling genes associated with root hair development. *Am. J. Bot.* 102, 21–35. doi:10.3732/ajb.1400458.

Lamesch, P., Berardini, T. Z., Li, D., Swarbreck, D., Wilks, C., Sasidharan, R., et al. (2012). The Arabidopsis Information Resource (TAIR): Improved gene annotation and new tools. *Nucleic Acids Res.* 40, D1202–D1210. doi:10.1093/nar/gkr1090.

Manzano, A. I., van Loon, J. J. W. A., Christianen, P. C. M., Gonzalez-Rubio, J. M., Medina, F. J., and Herranz, R. (2012). Gravitational and magnetic field variations synergize to cause subtle variations in the global transcriptional state of Arabidopsis in vitro callus cultures. *BMC Genomics* 13, 105. doi:10.1186/1471-2164-13-105.

Missirian, V., Conklin, P. A., Culligan, K. M., Huefner, N. D., and Britt, A. B. (2014). High atomic weight, high-energy radiation (HZE) induces transcriptional responses shared with conventional stresses in addition to a core “DSB” response specific to clastogenic treatments. *Front. Plant Sci.* 5, 364. doi:10.3389/fpls.2014.00364.

Obayashi, T., Aoki, Y., Tadaka, S., Kagaya, Y., and Kinoshita, K. (2018). ATTED-II in 2018: A Plant coexpression database based on investigation of the statistical property of the mutual rank index. *Plant Cell Physiol.* 59, e3. doi:10.1093/pcp/pcx191.

Palaniswamy, S. K., James, S., Sun, H., Lamb, R. S., Davuluri, R. V., and Grotewold, E. (2006). AGRIS and AtRegNet. A platform to link cis-regulatory elements and transcription factors into regulatory networks. *Plant Physiol.* 140, 818–829. doi:10.1104/pp.105.072280.

Papatheodorou, I., Fonseca, N. A., Keays, M., Tang, Y. A., Barrera, E., Bazant, W., et al. (2018). Expression Atlas: Gene and protein expression across multiple studies and organisms. *Nucleic Acids Res.* 46, D246–D251. doi:10.1093/nar/gkx1158.

Paul, A. L., Sng, N. J., Zupanska, A. K., Krishnamurthy, A., Schultz, E. R., and Ferl, R. J. (2017). Genetic dissection of the Arabidopsis spaceflight transcriptome: Are some responses dispensable for the physiological adaptation of plants to spaceflight? *PLoS One* 12, e0180186. doi:10.1371/journal.pone.0180186.

Paul, A. L., Zupanska, A. K., Ostrow, D. T., Zhang, Y., Sun, Y., Li, J. L., et al. (2012). Spaceflight transcriptomes: Unique responses to a novel environment. *Astrobiology* 12, 40–56. doi:10.1089/ast.2011.0696.

Paul, A. L., Zupanska, A. K., Schultz, E. R., and Ferl, R. J. (2013). Organ-specific remodeling of the Arabidopsis transcriptome in response to spaceflight. *BMC Plant Biol.* 13, 112. doi:10.1186/1471-2229-13-112.

Salmi, M. L., and Roux, S. J. (2008). Gene expression changes induced by space flight in single-cells of the fern *Ceratopteris richardii*. *Planta* 229, 151–159. doi:10.1007/s00425-008-0817-y.

Sugimoto, M., Oono, Y., Gusev, O., Matsumoto, T., Yazawa, T., Levinskikh, M. A., et al. (2014). Genome-wide expression analysis of reactive oxygen species gene network in Mizuna plants grown in long-term spaceflight. *BMC Plant Biol.* 14, 4. doi:10.1186/1471-2229-14-4.

Sullivan, A., Purohit, P. K., Freese, N. H., Pasha, A., Esteban, E., Waese, J., et al. (2019). An ‘ eFP ‐Seq Browser’ for visualizing and exploring RNA sequencing data. *Plant J.* Epub ahead. doi:10.1111/tpj.14468.

Szklarczyk, D., Gable, A. L., Lyon, D., Junge, A., Wyder, S., Huerta-Cepas, J., et al. (2019). STRING v11: protein–protein association networks with increased coverage, supporting functional discovery in genome-wide experimental datasets. *Nucleic Acids Res.* 47, D607–D613. doi:10.1093/nar/gky1131.

Toufighi, K., Brady, S. M., Austin, R., Ly, E., and Provart, N. J. (2005). The botany array resource: e-Northerns, expression angling, and promoter analyses. *Plant J.* 43, 153–163. doi:10.1111/j.1365-313X.2005.02437.x.

Vandenbrink, J. P., Herranz, R., Poehlman, William, L., Feltus, Frank, A., Ciska, M., Medina, Francisco, J., et al. (2019). RNA‐seq analyses of *Arabidopsis thaliana* seedlings after exposure to blue‐light phototropic stimuli in microgravity. *Am. J. Bot.* 106, 1466–1476.

Waese, J., Fan, J., Pasha, A., Yu, H., Fucile, G., Shi, R., et al. (2017). ePlant: Visualizing and exploring multiple levels of data for hypothesis generation in plant biology. *Plant Cell* 29, 1806–1821. doi:10.1105/tpc.17.00073.

Willems, P., Mhamdi, A., Stael, S., Storme, V., Kerchev, P., Noctor, G., et al. (2016). The ROS wheel: Refining ROS transcriptional footprints. *Plant Physiol.* 17, 1720–1733. doi:10.1104/pp.16.00420.

Zhou, M., Sng, N. J., Lefrois, C. E., Paul, A. L., and Ferl, R. J. (2019). Epigenomics in an extraterrestrial environment: Organ-specific alteration of DNA methylation and gene expression elicited by spaceflight in *Arabidopsis thaliana*. *BMC Genomics* 20, 205. doi:10.1186/s12864-019-5554-z.

Zupanska, A. K., Lefrois, C., Ferl, R. J., and Paul, A. L. (2019). HSFA2 functions in the physiological adaptation of undifferentiated plant cells to spaceflight. *Int. J. Mol. Sci.* 20, E390. doi:10.3390/ijms20020390.

Zupanska, A. K., Schultz, E. R., Yao, J., Sng, N. J., Zhou, M., Callaham, J. B., et al. (2017). ARG1 functions in the physiological adaptation of undifferentiated plant cells to spaceflight. *Astrobiology* 17, 1077–1111. doi:10.1089/ast.2016.1538.
